# Level of anxiety and results of psychomotor tests in young soccer players of different performance levels

**DOI:** 10.5114/biolsport.2022.106387

**Published:** 2021-07-23

**Authors:** Dominika M. Wilczyńska, Frank Abrahamsen, Agnieszka Popławska, Piotr Aschenbrenner, Marcin Dornowski

**Affiliations:** 1 Gdansk University of Physical Education and Sport, Social Science Department, Poland; 2 Norwegian School of Sport Sciences, Coaching and Psychology Department, Norway; 3 University of Social Sciences and Humanities, Sopot Campus, Poland; 4 Gdansk University of Physical Education and Sport, Biomechanics and Sport Engineering Department, Poland; 5 Gdansk University of Physical Education and Sport, Sport Department, Poland

**Keywords:** Emotions, Vienna Test System, Child Athletes, Soccer, Performance

## Abstract

The aim of the current study is to determine how the level of state and trait anxiety differs between youth athletes of different performance levels and furthermore whether there are correlations between performance levels and psychomotor variables in the selected tasks. A sample of 97 boys, aged 11–12 years, practising soccer represented two groups: A – high performance level and B – lower performance level. Participants completed a state and trait anxiety inventory and performed selected psychomotor tests. The analyses demonstrated that the higher the levels of anxiety were, the shorter was the response time and more accurate were the responses in selected psychomotor tests. For the whole group, r = -0.224, p < 0.05, and for group B, r = -0.333, p < 0.05. Moreover, the findings showed a moderator effect of level (group A vs B) on reaction time, which was almost significant in state anxiety and significant in trait anxiety. For group B, trait anxiety was negatively related to reaction time (b = -0.002, SE = 0.001, t = -2.93, p = .004, 95% CI [-0.004, -0.001]). This means that the higher the trait anxiety was, the shorter was the reaction time in group B, but there was no significant effect in group A. The results of the study confirmed the negative correlation between the trait and state anxiety and reaction time. The higher the anxiety was, the shorter was the response time of child soccer players. Future research should determine whether athletes’ performance levels do affect performance under stress and replicate the study with different samples such as girls and different sport disciplines.

## INTRODUCTION

William James wrote in his 1890 psychology textbook: “My experience is what I disagree with” [1]. Emotions are subjective states of the organism, triggering prioritization for the associated action programme, accompanied by somatic changes, expressions, and behaviours [2]. Emotions affect cognitive processes, and there are several varieties of these interactions, from the unconscious filtering of incoming information to the conscious preoccupation that occurs when we are worried about something [1].

The results of selected studies confirm our assumption that anxiety impacts the cognitive process of attention, i.e., the observation that anxiety narrows the scope of attention. High levels of anxiety cause the nervous system to change into a modality wherein attention is narrowed [1]. This phenomenon is traditionally associated with the Easterbrook hypothesis, which states that physiological arousal leads to attention narrowing or tunnel vision that impacts cognition [2], potentially resulting in a more intensive focus on the task and a faster reaction time. Furthermore, the broaden-and-build theory of positive emotions by Fredrickson [3] proposes that negative emotions narrow people’s ideas about possible actions, whereas positive emotions do the opposite, broadening people’s ideas about possible actions and creating awareness of a wider range of thoughts and actions. Carver and Scheier [4] also write in their study that anxiety represents an interrupter of action and a call for reconsideration of what goals deserve the person’s most immediate attention and effort. Further complicating the interplay between anxiety, actions, and performance is the notion that there exist individual zones of optimal functioning (IZOF) [5], suggesting that athletes can achieve good results even when exhibiting high levels of anxiety. Consequently, anxiety should be considered individually, as every athlete may have an IZOF where they operate most effectively. Moreover, the interpretation of the symptoms of athletes’ anxiety is also highly important. Jones and Hanton [6] developed a model that follows the work of Carver and Scheier [4], in which an individual’s anxiety can have both positive and negative effects on the effectiveness of an action depending on his or her confidence in being able to deal with it. Similarly, Eriksen et al. [7] in the cognitive activation theory of stress (CATS) underline that the stress response could depend on acquired expectancies of the outcome of the stimulus and of the specific responses available for coping.

Considering children’s sport participation and sport performance, many studies have focused on the influence of parents and coaches on children’s performance anxiety [8–10], while a few have considered the influence of the level of anxiety on performance. In a qualitative study, Gould et al. [11] reported that most young athletes are not under excessive stress. Rather, certain children in specific situations experience high levels of competitive state anxiety. This confirms the cognitive approach, underlining the significant role of the assessment of the situation and the emotional reaction that follows that assessment and influences behaviour [12]. Pierpaolo and Antonia [13] underline that emotional arousal management as anxiety and stress is one of the primary mental abilities that strengthen support or inhibit sports performance. The performance of high-level athletes is related to coping with emotional arousal and is crucial to implement among junior athletes to achieve similar results in terms of performance. The aspect of anxiety belongs to all athletes, making it one of the essential sports psychology topics. Therefore, investigating the level of concern among children and teenagers practising sport appears to be very reasonable. When children and youths experience high physiological arousal, which increases cognitive anxiety during the performance, they cannot meet their potential and thrive in the sport context [14]. Moreover, young athletes need special mental and emotional control training since their emotions are periodically unstable due to developmental aspects and immature defence mechanisms and coping strategies [11].The current study aimed to investigate the level of state and trait anxiety among young soccer players depending on their performance level and how state and trait anxiety influence performance. The study was carried out in laboratory conditions in which the psychomotor and cognitive abilities of young soccer players were examined using selected tests from the Vienna Test System (VTS). A review of studies [15] on the VTS demonstrated that it is a very useful objective measure of various psychological constructs and can be used to complement existing subjective measures in the field.

The examination of children’s emotionality through the assessment of anxiety in combination with a psychomotor test in laboratory conditions seems sensible. The conditions of the study allow us to test emotions during the performance of a moderate task and a more complicated (complex) task. As mentioned, the aim of the study is to determine how the level of state and trait anxiety differs between players of different sport/performance levels and, furthermore, whether there are correlations between performance levels and psychomotor variables in the selected tasks. Furthermore, we decided to focus on measuring trait anxiety as well, since this personality feature leads, to a small or large extent, to the impediment of mental forces and mechanisms by which school and sport performances are achieved [16]. As the traits can be modified in childhood and adolescence, the level of trait anxiety among children should be under observation.

## MATERIALS AND METHODS

### Participants

The data in this study were obtained from boys (N = 97, age 11–12 years) who practised soccer. The players were divided into two groups based on their sports level: level A, leading clubs that perform well in first regional junior leagues, and level B, clubs competing outside of the first regional junior league. Level A consisted of Lechia Gdansk and Gedania Gdansk representants (Pomeranian clubs) – the first league in the junior category. Level B was represented by boys from Salos Elk (Warmian-Masurian club) and two Pomeranian clubs: Kolbudy Soccer Academy and Sokol Euganowo – outside of the first league in the junior category. The descriptive statistics of the groups are presented in Table 1.

**Table 1:** Descriptive statistics

	Level A (N = 43)				Level B (N = 54)			
	x	SD	min	max	x	SD	min	max
Age [years]	11.6	0.5	11.0	12.0	11.6	0.6	11.0	13.0
Grade average	4.9	0.8	3.1	6.0	4.8	0.6	3.8	6.0
Number of trainings	3.4	0.5	3.0	4.0	3.1	0.3	3.0	4.0
Years of training	4.2	0.8	3.0	5.0	4.2	0.7	3.0	5.0

Both groups were homogeneous in terms of the described parameters, and there were no statistically significant differences between the groups when tested with Student’s t-test at a significance level of p < 0.05. In the level B group, the majority of boys (93%) lived in a small town/countryside. In the level A group, the players resided in large cities (84%) and small towns (16%).

### Study design and procedure

The Pomeranian Sport Federation and the Warmian-Masurian Sport Federation were informed about the project of the study. Coaches from 15 teams volunteered to participate in the study (6 teams representing level A and 9 clubs representing level B). Eventually, five clubs remained in the study. Ten clubs discontinued participation because of organizational issues.

The children participated in the study at the Laboratory of Physical Exertion between 9 o’clock and 12 o’clock in the morning. First, they completed the STAI-C (State Trait Anxiety Inventory-Children) and the 2 Vienna Test System (VTS) tests. Before commencing, the children were seated comfortably in a chair adjusted to their size and needs and in front of a console to perform the Reaction Time (RT) test and then the Determination Test for Kids (DTKI) sequentially.

### Methods

#### Measurement of state and trait anxiety

STAI-C, created by Spielberger et al. [17] consists of two 20-item scales that measure state and trait anxiety in children between the ages of 10 and 14. The A-State scale examines anxiety that is specific to the situation and prompts children to rate 20 statements from “hardly ever true” to “often true”. The A-Trait scale measures longer-term trait anxiety and addresses how the child typically feels. A separate score is produced for both scales. The reliability analysis indicated statistically significant reliability for psychological instruments STAI-C1 (state anxiety; Cronbach’s alpha: 0.894) and STAI-C2 (trait anxiety; Cronbach’s alpha: 0.946).

#### Measurement of psychomotor and cognitive abilities

The Vienna Test System (VTS) is a computerized psychological assessment tool that provides a reliable means of measuring ability and cognitive skills. We used the Determination Test for Children (DTKI) from the VTS in the current study. The test requires the respondent to use his or her cognitive skills, such as attention, short-term memory, and reaction time, in tasks that demand fast and accurate responses to changing visual and auditory stimuli. The children performing the DTKI test reacted to seven different visual and two different auditory stimuli [18, 19].

The Reaction Time test (RT) measured the multifaceted reaction time (seconds) of the children. The device generated visual stimuli that appeared on the monitor and auditory stimuli that were presented through earphones [19].

### Data analysis

The results of the tests were subjected to standard statistical analysis in the IBM SPSS Statistics 26.0.0.1 software package. After checking the normality of the distributions, basic descriptive statistics were calculated, such as the mean, median, standard deviation, minimum, and maximum. Differences in the mean values for parametric data between groups were analysed by the t-test for independent groups. The interdependencies between variables in particular groups were assessed using correlation analysis (Pearson’s r) and moderation analysis. The reliability of the tests was assessed by calculating Cronbach’s alpha.

### Ethics

The procedures of the following study were in accordance with the Helsinki Declaration and both parents of the participants and the participants themselves signed an informed consent form. The document number from the bioethics committee is KB-13/17.

## RESULTS

The t-test for independent groups showed a significant difference in the level of state anxiety results in Table 2; the level A group obtained lower results compared to the level B group; M = 36.52, SD = 9.16 vs. M = 31.83, SD = 8.97; t95 = 2.53; p < 0.05.

**Table 2:** Results of the STAI-C1 (state anxiety) and STAI-C2 (trait anxiety) instruments.

	Level A (N = 43)				Level B (N = 43)			
	x	SD	min	max	x	SD	min	max
State anxiety	31.8*	8.8	20.0	49.0	36.5*	9.1	20.0	52.0
Trait anxiety	39.2	12.2	20.0	58.0	40.4	11.0	21.0	58.0
Trait anxiety quartiles	2.5	1.2	1.0	4.0	2.6	1.0	1.0	4.0
State anxiety quartiles	2.3	1.1	1.0	4.0	2.7	1.1	1.0	4.0

* Statistically significant. p < 0.05

The results of the DTKI test (Table 3) showed no significant differences between the groups. The statistics for the correct responses (n) were M = 201.1, SD = 27.2 for the level B group and M = 204, SD = 22.3 for the level A group. The statistics for the incorrect responses (n) were M = 16, SD = 11.1 for the level B group and M = 17.4, SD = 10.6 for the level A group.

**Table 3:** Results of the *Determination Test for Children* of the Vienna Test System.

	Level A (N = 43)				Level B (N = 54)			
	x	SD	min	max	x	SD	min	max
Correct responses (n)	204.0	22.3	140.0	250.0	201.1	27.2	141.0	288.0
Incorrect responses (n)	17.4	10.6	1.0	42.0	16.0	11.1	1.0	58.0
Omitted responses (n)	21.6	8.1	6.0	39.0	20.9	9.2	6.0	51.0

There were also no significant differences between level A and level B in the RT test (results in Table 4). Both groups achieved similar results for reaction time (s) (level B: M = 458; SD = 79; level A: M = 256; SD = 51).

**Table 4:** Results of the *Reaction Time* test of the Vienna Test System

	Level A (N = 43)				Level B (N = 54)			
	x	SD	min	max	x	SD	min	max
Reaction time (s)	456.4	51.1	348.0	567.0	458.3	79.2	319.0	778.0
Motor time (s)	173.8	47.4	60.0	292.0	179.4	44.8	77.0	289.0
Reaction time _log	2.7	0.0	2.5	2.8	2.7	0.1	2.5	2.9

To examine the relationship between anxiety and the psychomotor and cognitive variables, correlation analyses were performed for the groups. First, correlations were computed for level A and level B separately and then for both groups combined (whole group). Negative correlations were found between state anxiety and reaction time (s) for the whole group (r = -0.224, p < 0.05) and for the level B group (r = -0.333, p < 0.05), which means that the higher state anxiety was, the lower was the reaction time is. State anxiety was positively correlated with correct responses (n); the higher the anxiety was, the higher was the number of correct responses (n) (whole group: r = 0.299, p < 0.01; level A: r = 0.378, p < 0.05; level B: r = 0.291, p < 0.05). Trait anxiety was negatively correlated with reaction time (s) and positively correlated with correct responses (n). The higher the trait anxiety was, the lower was the reaction time (whole group: r = -0.209, p < 0.05; level B: r = -0.343, p < 0.05), and the higher the trait anxiety was, the higher was the number of correct responses (whole group: r = 0.286, p < 0.01; level B: r = 0.339, p < 0.05). It should be noted that for the level A group, anxiety was correlated with one variable only, the correct response rate.

**Fig. 1:**
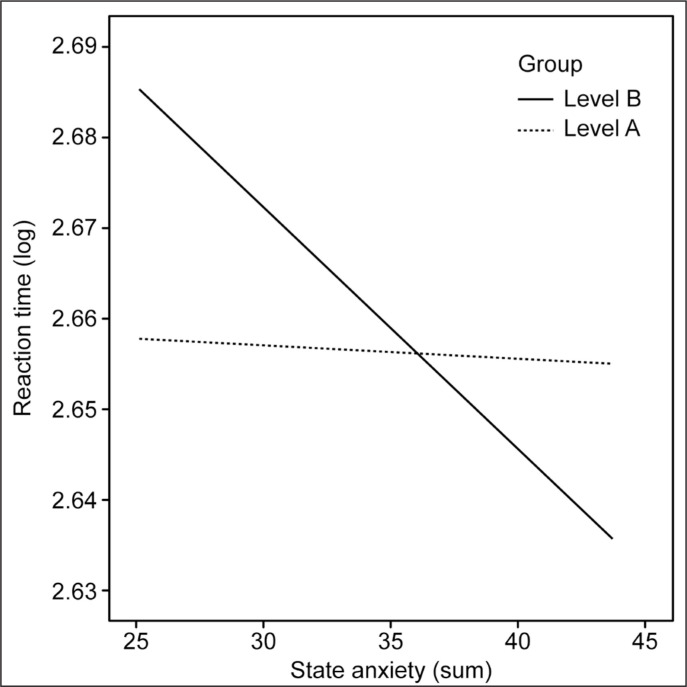
Trend lines of the correlation between moderation of state anxiety and reaction time (RT test).

The next step of the analysis was to assess the impact of the sport level as a moderator of the relationship between reaction time (RT S4) and state and trait anxiety. In the first step of the analysis, the reaction time was the dependent variable, state anxiety was the independent variable, and the moderator was level A vs level B. It was found that the model was significant, the influence of the independent variable was also significant, and the interaction with the moderator was on the verge of significance. In the case of the level B group, the higher the state anxiety, the shorter the reaction time was. For the level A group, there was no such relationship. The results indicated that the reaction time could be predicted by state anxiety (b = -0.01, SE = 0.002, t = -2.45, p = .02, 95% CI [-0.01, -0.001]). The interaction effect almost reached significance for the level B group (b = 0.003, SE = 0.001, t = 1.8, p = .08, 95% CI [-0.00, -0.01]). For the level B group, anxiety was negatively related with reaction time (b = -0.003, SE = 0.001, t = -2.91, p = 0.005, 95% CI [-0.005, -0.001]). There was no such effect for the level A group (b = -0,0002, SE = 0.001, t = -0.15, p = .88, 95% CI [-0.002, 0.002]). The results are presented in Figure 1.

**Fig. 2:**
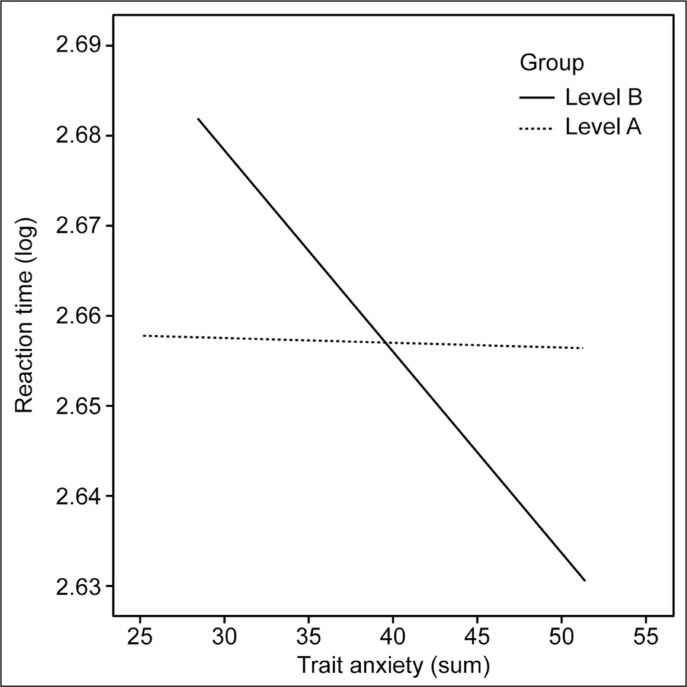
Trend lines of the correlation between moderation trait anxiety – reaction time (RT test).

In the second stage of the statistical analysis, the reaction time was again the dependent variable, trait anxiety was the independent variable, and the moderator was level A vs level B. The analysis revealed a significant result, where the influence of the independent variable and an interaction with the moderator were found. In the case of the level B group, the higher the trait anxiety was, the faster was the reaction time. There was no such relationship in the level A group. Based on the analysis of moderation, we can conclude that the reaction time of the RT test can be predicted by trait anxiety (b = -0.004, SE = 0.002, t = -2.59, p = .01, 95% CI [-0.008, -0,001]) and by club (b = -0.09, SE = 0.05, t = -1.94, p = .05, 95% CI [-0.17, 0.002]), and the interaction effect with the moderator was significant: b = 0.002, SE = 0.001, t = 2.03, p = .04, 95%, CI [0.00, 0.04]. For group B, anxiety was negatively related to reaction time (b = -0.002, SE = 0.001, t = -2.93, p = .004, 95% CI [-0.004, -0.001]). There was no such effect for the level A group (b = -0.00, SE = 0.001, t = -0.14, p = .97, 95% CI [-0.002, 0.002]). The results are presented in Figure 2.

## DISCUSSION

The aim of the current study was to examine state and trait anxiety in young soccer players and how it and the players’ performance levels affected performance in the psychomotor tests from the Vienna Test System. For these tests, higher levels of anxiety (both state and trait) were correlated with shorter response times and more correct responses, which is in line with the hypothesis of attentional narrowing and changes in the nervous system (becoming more alert). Isolating the state anxiety results, one could argue that the test by itself is not stressful enough to evoke strong emotions (i.e., anxiety) that might impair performance. In the present investigation, we did not conduct a manipulation check, which is a limitation. In the future, we would like to include manipulation checking when replicating the study and must redesign the experiment in order to do so. The manipulation of stress needs to be taken seriously, especially for children, to avoid unethical practices. This was of course no concern here, but elevating stress in participants needs to be both viable and ethically handled, which will limit the induced state anxiety levels in future experiments. Notably, however, the trait anxiety results were similar to those of state anxiety, and for that reason, we would consider the findings for our two samples to be reliable. As mentioned above, replicating the study is important, preferably with different samples such as girls and different sports. Grossbard et al. [20] in a study on male and female tennis and basketball youth players highlighted the gender differences in anxiety in association with social desirability and goal orientation. Performance anxiety was negatively correlated with social desirability only in girls, while all the indices of performance anxiety in boys were negatively related to task orientation.

One of the important distinctions of the findings in the present study was the difference between the level A group (leading clubs that perform well in the first football league in junior category) and the level B group (clubs that participate in lower-level, local competitions, outside of the first league). This was a small sample, so any inferences should be made cautiously; furthermore, the transferability between sport level, sport performance and performance in the VTS is not part of this study. However, the moderator effects of level (group A vs B) on reaction time (almost significant in state anxiety and significant in trait anxiety) were in the same direction for both state and trait anxiety: the higher the anxiety was, the shorter was the reaction time in the level B group, while no effect was observed for the level A group. The cognitive activation theory of stress (CATS) [7] explains the potential training effect of stress exposure. That one group, A, experienced no reduction in reaction time at higher levels of anxiety is interesting; could this be a training effect (that the players performed more consistently) from being exposed to a higher competition level and the subsequent stress? Studies on high-level youth pistol shooters revealed that longer quiet-eye duration, which has been associated with more successful performance and less deterioration in skills, was shorter during anxiety conditions. This result suggests that narrow and external attention in shooters can be disturbed by arousal [21]. Future studies should determine whether athletes’ performance levels affect performance under stress, but research has shown that more competitive athletes interpret their anxiety symptoms as more facilitative for their performance than their less competitive peers [22]. Research has also provided evidence that this interpretation of anxiety might be trainable [23]. Hanton and Connaughton [24] suggested that the reason for this was that anxiety symptoms perceived to be under athletes’ control were reported as more facilitative for performance than those not under their control. Furthermore, these researchers reported how increases or decreases in self-confidence affected perceived performance accordingly. Linking laboratory research with research from ecologically valid settings in the future will be important to determine whether the “skills” translate from the laboratory to the soccer field, as there is, as Ericsson and Williams [25] argue, no assurance that performance on standardized tasks will elicit the same performance elsewhere. On the other hand, MacCarthy et al. [26] studied youth sports athletes, finding that anxiety correlated positively with interfering thoughts and concentration disturbance during the competition. In contrast, the effects of anxiety before the competition were associated with thoughts of escape. These findings demonstrate how cognitive interference caused by anxiety impacts the performance in youth sport.

To summarize, it is worth pointing out that research does not suggest that emotions with high, positive valence and low, negative valence are always necessary for performance success. There are situations in which positive affect, negative affect, or awareness of an emotional state would not be ideal or even appropriate and may impair processes that support performance. An example could be the experience of a flow state, linked to optimal performance and optimal experiences, where performers depict being fully absorbed in the activity and being in the present, rather than being aware of particular emotions [27]. However, the anxiety could benefit performance through narrowing the attention or increasing the effort, and we have many avenues of research to support this statement [24, 28, 29]. In past research, such as the above, it has been reported that handling stress is trainable and that athletes might learn to interpret anxiety symptoms as helpful for an upcoming task (at least up to a point). Thus, the question is not whether young athletes should train to cope with stress and adversity but rather how and when to train. We realize that our study is not a novelty. However, we firmly believe that performing research to control, observe, and measure anxiety in children can bring new ideas on preparing children for sports performance and life adversities mentally.

## CONCLUSIONS

In the present study, we conducted a detailed investigation with young soccer players from two performance level groups that differed in how they handled psychological tasks. The young athletes from level B are characterized by a higher level of trait anxiety and, therefore, a shorter reaction time. The same tendency was also observed for level B players in state anxiety. Future research with youth sports could yield simple experiments within an ecologically valid setting to examine similar issues.
